# Emergency Laparotomy in the Critically Ill: Futility at the Bedside

**DOI:** 10.1155/2018/6398917

**Published:** 2018-08-26

**Authors:** Niels D. Martin, Sagar P. Patel, Kristen Chreiman, Jose L. Pascual, Benjamin Braslow, Patrick M. Reilly, Lewis J. Kaplan

**Affiliations:** Division of Traumatology, Surgical Critical Care & Emergency Surgery, Department of Surgery, Perelman School of Medicine at the University of Pennsylvania, Philadelphia, PA, USA

## Abstract

**Background:**

Critically ill patients are often evaluated for an intra-abdominal catastrophe. In the absence of a preoperative diagnosis, abdominal exploration may be offered despite desperate circumstances. We hypothesize that (1) abdominal exploration for such patients is associated with a high mortality and (2) commonly obtained physiologic measures at laparotomy anticipate mortality.

**Methods:**

All acute care surgery (ACS) patients undergoing emergency laparotomy at a quaternary referral center during a 3-year period were reviewed. Inclusion was defined by emergency laparotomy in the operating room (OR) in a patient with an American Society of Anesthesiologists (ASA) score ≥4 or bedside laparotomy in the ICU (BSL). Mortality was the primary endpoint and was stratified by demographics, admitting service, surgical findings, and physiology. Comparisons between OR and BSL were by Fisher's exact and Mann–Whitney tests.

**Results:**

144 patients underwent emergency laparotomy (45 BSL vs. 99 OR). Overall mortality was 55.6% (77.8% BSL vs. 45.5% OR; *p* < 0.001). Mortality by admitting service was cardiac 71.4% (*n*=42), medical 70% (*n*=30), ACS 42% (*n*=50), and other 36.4% (*n*=22) services. Preoperative lactate levels were higher in nonsurvivors (2.7 vs. 8.5 mmol/L, *p* < 0.001), as was vasopressor use (62.5% vs. 97.5%, *p* < 0.001), acute kidney injury (51.6% vs. 72.5%, *p* < 0.01), leukocytosis (53.1% vs. 71.3%, *p* < 0.04), and anemia (45.3% vs. 71.3%, *p* < 0.01). The presence of any identifiable abdominal pathology established a 90% mortality rate.

**Conclusions:**

The need for BSL portends an extremely high mortality rate and is likely useful in preintervention counselling. Emergency OR laparotomy leads to mortality in nearly half of such patients and is anticipatable based on concurrent abnormal physiology.

## 1. Background

An intra-abdominal catastrophe may be suspected when intensive care unit (ICU) patients demonstrate rapidly progressive critical illness without an alternative diagnosis. In this setting, surgical decision-making may be limited by the absence of a reliable physical examination secondary to an altered sensorium compounded by physiologic instability rendering the patient too unstable to travel for advanced imaging. In these cases, emergent laparotomy may be offered regardless of age, despite an anticipated high mortality [1–3]. While potentially lifesaving in a select few, emergent surgery in the OR or at the bedside utilizes substantial institutional resources, often results in nonbeneficial outcomes for which there has been insufficient time to explore prior to intervention, and potentially provides false hope to the patient's family or significant other(s). The compressed timeframe between consultation and intervention is commonly complicated by a lack of knowledge of the patient's goals-of-care desires within the context of an unexpected and acutely life-threatening critical illness [4, 5]. Additionally, the lack of a preexisting therapeutic relationship between the newly consulted surgeon and the patient and the patient's decision makers lays the foundation for a contested dynamic in the event of an untoward outcome [6].

Outcomes for urgent surgery appear to be worse compared to similar elective operations [7]. Emergency surgery in the most critically ill patients, in the OR or at the bedside, by comparison remains less well explored. Accordingly, we sought to evaluate outcomes of severely critically ill patients who underwent an undirected emergency exploratory laparotomy. We hypothesized that (1) the ability to transport the patient to the OR defined a population with a lesser mortality than those undergoing bedside exploration and (2) mortality would strongly correlate with commonly identified physiologic derangements and abnormal laboratory data.

## 2. Methods

This study was performed by a retrospective analysis of a prospectively collected, institutional registry of an Acute Care Surgery (ACS) service. This service captures all emergency general surgery consults in a quaternary referral, adult, urban, and academic medical center that houses 772 acute care beds and manages nearly 40,000 annual admissions. This study was performed with approval of the local institutional review board. The registry itself is proprietary to the institution and is populated manually, after discharge, by trained registrars not associated with the clinical team. The ACS service has 24/7 OR access, but also maintains a mobile cart of materials appropriate for emergency bedside laparotomy for those too ill for transport.

Patients were selected from the registry on the basis of the following defining characteristics: (1) emergency laparotomy in the OR and an American Society of Anesthesiologist (ASA) physical status classification score was 4 (severe systemic disease, i.e., a constant threat to life such as sepsis, ongoing myocardial ischemia, or DIC) or 5 (moribund and is not expected to survive without operation) and (2) emergency laparotomy at the bedside (BSL). The registry was queried over a 37-month period (June 2012–June 2015). Patients were excluded for nonemergency laparotomy or planned reexploration regardless of procedure location.

Patient demographics including age, gender, and length of stay, along with physiological parameters captured immediately prior to the time of surgical intervention were extracted from the electronic medical record. The physiologic parameters included serum lactate, vasopressor infusion, acute kidney injury (defined as RIFLE “I”), leukocytosis (WBC outside the lab reference range), and anemia (Hgb < lab reference range). Patients were categorized based on the ICU in which they were cared as this corresponded to their admitting service at our institution. These locations included the Cardiothoracic (CT) ICU, the Medical ICU, and the Surgical ICU (further refined as patients on the ACS service vs. all other surgical services). Surgical findings were categorized into the following groups: abdominal compartment syndrome, colitis, global intestinal ischemia, segmental intestinal ischemia, hemorrhage, intra-abdominal sepsis with or without perforation (peritonitis), and no intra-abdominal findings.

Inpatient mortality was stratified by procedure location, ICU, and findings at laparotomy. Intergroup comparisons were by Fisher's exact test for categorical variables and Mann–Whitney test for continuous data. Statistical analysis was performed using SPSS Statistics (IBM Inc., New York,) with significance assumed for *p* < 0.05.

## 3. Results

### 3.1. Entire Cohort

**Figure 1:**
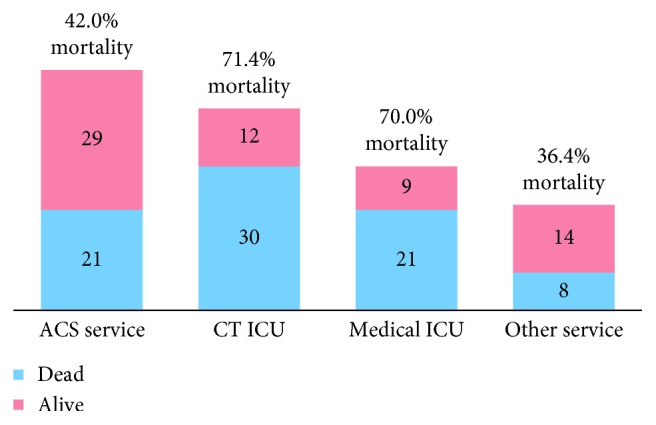
Mortality rates by primary consulting service.

144 patients met inclusion criteria. The average age was 62.9 ± 14.9 years with 99 (69%) OR laparotomies and 45 (31%) BSL. Overall in-hospital mortality was 55.6% (77.8% BSL vs. 45.5% OR, *p* < 0.001). Mortality by admitting service was cardiac 71.4% (*n*=42), medical 70% (*n*=30), ACS 42% (*n*=50), and other 36.4% (*n*=22) services (Figure 1). Patients admitted to the ACS service either presented to the hospital with severe critical illness or became so after admission for an emergency general surgery diagnosis.

### 3.2. OR Laparotomy

**Figure 2:**
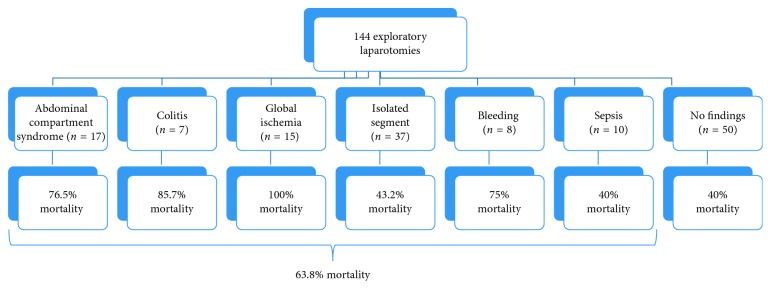
Mortality rates for all operative finding types.

Abdominal pathology was identified in 94 (65.3%) patients; 50 (34.7%) patients had no identifiable pathology on exploration (Figure 2). Any abdominal pathology defined a mortality rate of 63.8% while no discernable pathology foretold a more favorable mortality rate of 40% (*p* < 0.01 vs. abdominal pathology). Identified pathology included abdominal compartment syndrome (17 and 18.1%), colitis (7 and 7.4%), global intestinal ischemia (15 and 16.0%), segmental intestinal ischemia (37 and 39.4%), intra-abdominal hemorrhage (8 and 8.5%), and peritonitis (10 and 10.6%). The mortality rate by identified pathology ranged from 40% for peritonitis to 100% for those with global intestinal ischemia (Figure 2).

Significant physiologic differences at the time of laparotomy were found between survivors and nonsurvivors on univariate analysis (Table 1); since mechanical ventilation was required in all patients, its presence was not associated with increased mortality in this critically ill cohort. Preoperative lactate levels were higher in nonsurvivors (8.5 vs. 2.7 mmol/L, *p* < 0.001), as was vasopressor use (97.5% vs. 62.5%, *p* < 0.001), RIFLE class “I” acute kidney injury (72.5% vs. 51.6%, *p* < 0.01), leukocytosis (71.3% vs. 53.1%, *p* < 0.04), and anemia (71.3% vs. 45.3%, *p* < 0.01).

### 3.3. Bedside Laparotomy

**Figure 3:**
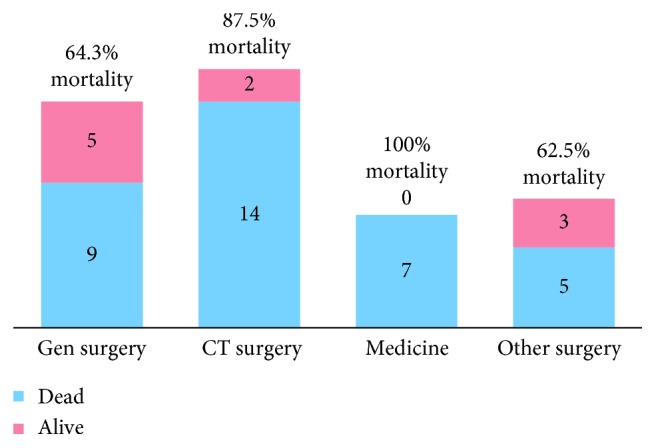
Mortality rate by primary service for bedside laparotomies.

**Figure 4:**
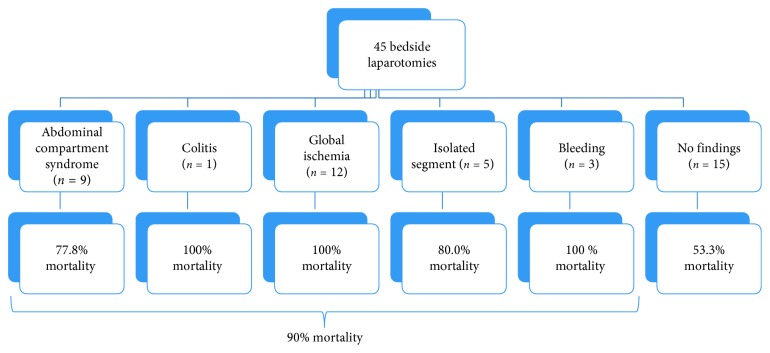
Mortality rates for all operative finding types in bedside laparotomies.

No BSL patient was able to sign their own consent. Mortality (Figure 3) was highest in those in the MICU (7/7, 100%) followed by those in the CI-ICU (14/16; 87.5%), and was similar for the two groups in the SICU, ACS (9/14; 64.3%) and other (5/8; 62.5%). Abdominal pathology was identified in 30 (66.7%) BSL patients, whereas 15 (33.3%) BSL patients had no intra-abdominal findings (Figure 4). In BSL patients, mortality was 90.0% with any intra-abdominal pathology but only 53.3% in those without identified pathology (*p* < 0.01). Identified surgical pathology included abdominal compartment syndrome (*n*=9, 30.0%), colitis (1, 3.3%), global intestinal ischemia (12, 40.0%), segmental intestinal ischemia (5, 16.7%), and intra-abdominal hemorrhage (3, 10.0%), with mortality rates ranging from 77.8% to 100%.

## 4. Discussion

Determining the presence of surgically correctable causes of severe critical illness may be quite challenging when common diagnostic aids such as advanced imaging are precluded due to hemodynamic instability. While portable CT scanning is used most commonly for brain imaging, it is not universally available and is challenged with imaging quality that is less than desirable for abdominal imaging. Accordingly, abdominal exploration is often considered as both diagnostic and potentially therapeutic for those with acute severe critical illness without a well-defined alternate etiology. Hemodynamically unstable patients who are unsafe to transport to the OR for whom an intra-abdominal catastrophe is believed to be the underpinning etiology may be explored at the bedside in the ICU [8, 9]. Unsurprisingly, such patients are believed to have a rather poor outcome either with or without intervention, leaving exploration as an “intervention of last resort.” There is no well-defined metric by which the postoperative outcome may be predicted with sufficient certainty to inform surrogate decision makers, as well as the surgeon, in deciding on the advisability of undertaking bedside exploration with regard to outcome and quality of life.

Since the BSL group is similar to, but distinct from, those stable enough for transport to the OR, this study is important in that it is the largest direct comparison of these two challenging groups of patients faced by Acute Care Surgeons. Unsurprisingly, we documented a high mortality rate in both groups, and as hypothesized, the highest mortality was in those requiring BSL due to instability. While seemingly intuitively obvious, the value of this observation lies in its ability to more precisely quantify an objective risk-benefit analysis for patients with severe acute critical illness who are being considered for abdominal exploration. While the “*n*” of the group is relatively small, it is reasonable to assume that our patients are similar to those in other institutions since they were heterogeneously distributed over several different ICUs and services, as opposed to being a homogeneous cohort of liquid organ transplant patients for example.

The extremely high mortality in those undergoing BSL suggests that this dire prognosis is underappreciated by both consulting physicians and Acute Care Surgeons alike. Alternatively, the grim outcome may be recognized but exploration remains a viable option, however unlikely, because futility has not been clinically reached either. Both of these propositions present an opportunity for improved communication to clearly outline the high likelihood of nonbeneficial outcomes including but not limited to death in an acute care facility, prolonged ICU length of stay, persistent organ failure, death in a long-term care facility, and failed obligations [10].

We believe that our data will be useful in providing appropriate information to all stakeholders in the decision-making process regarding BSL. Implicit in this approach is the ability to understand the patient's goals for intervention, if they were able to articulate them to the team via their surrogate decision maker. Such autonomy and substituted judgment, respectively, are cornerstones of a patient and family centered approach to inpatient care. When the surrogate is bereft of the specific knowledge to relate the patient's desires, decision-making is quite challenging and often devolves to an approach that embraces all that is medically feasible even if it is not necessarily able to restore premorbid level of function in a predictable fashion [11]. Indeed, in many circumstances, including those noted in this study, survival is not a reasonable goal for nearly all too unstable to reach the OR for laparotomy [6, 12, 13].

In a more nuanced way, even if survival was able to be reasonably achieved, what is required to achieve that goal may not be acceptable to some patients. Such discussions more often occur in the context of inexorably progressive medical conditions such as COPD, malignancy, and heart failure. Acute severe critical illness often occurs without such discussions having occurred and leaves surrogates and clinicians without clear guidance, perhaps leading to highly mortal bedside exploration including in a subset without identified intra-abdominal pathology. Guidelines, clinical pathways, multiprofessional team rounds including family members or surrogates have all been suggested as viable means of facilitating decision-making and communication [14]. Since surgery without a beneficial outcome engenders significant financial, resource, and personnel costs, such activity often invites careful scrutiny at the local hospital or larger system level [15]. Such activities are increasingly likely to be met with external guidance with the genesis of critical care organizations that are horizontally integrated across a single system.

We found differences in outcome based upon the index indication for admission as determined by primary service, especially for those undergoing BSL. Further, the types of pathologies ultimately found on exploration resulted in different mortality rates. The highest survival rates in both the OR and BSL cohorts occurred when no intra-abdominal pathology was found, albeit at the cost of additional stress. Any pathologic findings at BSL were associated with an extremely high mortality rate. Even the ideal emergency resection procedure at the bedside—isolated segmental intestinal ischemia—foretold an 80% mortality rate which was closely followed by a 77.8% mortality rate for abdominal compartment syndrome. Most likely, the low survivorship in this select group reflects the underlying comorbid conditions as well as the impact of the index cause for admission augmented by surgical stress. Indeed, both of these surgically correctable conditions often necessitate repeat exploration in the OR or at the bedside.

Mortality-impacting physiologic factors included preoperative serum lactate, vasopressor infusion, acute kidney injury, leukocytosis, and preoperative anemia. These elements may be best used as additional data points highlighting the likelihood of nonbeneficial outcomes or as triggers for either a goals-of-care discussion or palliative care consultation. Common triggers for palliative care consultation in the ICU have been articulated but may not specifically include the patient with severe acute critical illness, especially as all diagnostic and therapeutic options may not be perceived as being either exhausted or inappropriate [16]. Expanding the opportunities for palliative care collaboration and perhaps limiting surgical consultation for operative management of those with anticipated nonbeneficial outcomes appears warranted on the basis of our data as well as explorations of the typography of communication failure around surgical interventions [17].

This study has relevant limitations that may influence the applicability and generalizability of our findings. As a retrospective, single institution outcomes assessment, it may only represent local practice patterns and therefore local outcomes. Based on practice patterns, the clinical decision for OR compared to BSL introduces a selection bias as perceived illness severity likely informs operative location decision-making. However, transport out of the ICU is a highly dangerous event for the critically ill and is likely to guide risk/benefit assessments. We did not collect what informed this decision but all ACS staff also serve as intensivists, limiting some variability in decision-making. Further, because we only focused on in-hospital mortality, long-term outcomes in survivors, including quality of life, functional outcomes, readmission, and care recidivism, were not assessed. Similarly, morbidity after operation including infections, organ failures, and discharge destination was not tracked, but represents important investigative domains that may also inform the decision-making process. We also did not assess the hospital charges in the patient cohorts, nor the time spent in providing care—important components of resource prediction and workload planning that would be appropriate to assess in a prospective fashion, especially since the in-house Acute Care Surgeon is often multiply tasked, especially at night [18].

Future research in this area should also include the consideration for bedside laparoscopy as has been described by Alemanno et al. [19]. Although not performed in our current dataset, with the appropriate resources at the bedside, laparoscopy may be a less invasion method of assessment. It would require additional equipment and likely carries increased cost.

Perhaps most importantly, the database did not include patients for whom ACS was consulted but for whom operation was not offered. While important, the focus of this study was those in whom operation was offered and whether procedure location defined distinct populations with different outcomes.

It should be noted that the authors do feel that some patients will still warrant bedside laparotomy. The intended purpose of this manuscript is not end bedside explorations but to give the practicing surgeon an objective perspective on this cohort along with some prognostic factors that can be weighed during goals-of-care conversations and futility assessments.

## 5. Conclusion

Outcomes of abdominal explorations of patients with severe acute critical illness are poor, especially when patients are hemodynamically unstable and cannot undergo exploration in the OR. Attention should be given to the underlying pathology by referring service line, along with current physiology when formulating surgical options and considering the advisability of operative intervention in a cohort for whom abdominal pathology is not clearly established; expanded triggers for palliative care consultation may derive from these physiologic and laboratory variables. This information may inform goals-of-care discussions with proxy decision makers, surgical decision-making, and further enhance patient- and family-centered care initiatives.

## Figures and Tables

**Table 1:** Patient demographics and preoperative physiology versus mortality.

	All emergent laparotomies			Bedside laparotomies		
	Deaths (*n*=80)	Survivors (*n*=64)	*p* value	Deaths (*n*=35)	Survivors (*n*=10)	*p* value
Age, years (SD)	64.35 (14.2)	61.2 (15.6)	0.2069	65.1 (14.75)	56.4 (15.65)	0.1117
Lactate, mmol/L (SD)	8.54 (6.463)	2.716 (3.08)	**0.0001**	10.66 (6.897)	2.35 (1.074)	**0.0005**
Vasopressor, *n* (%)	78 (97.5%)	40 (62.5%)	**0.0001**	34 (97.14%)	8 (80%)	0.1195
Acute renal failure	58 (72.5%)	33 (51.6%)	**0.0146**	28 (80%)	6 (60%)	0.2279
Leukocytosis	57 (71.25%)	34 (53.1%)	**0.0365**	28 (80%)	6 (60%)	0.2279
Low hemoglobin	57 (71.25%)	29 (45.3%)	**0.0021**	26 (74.28%)	5 (50%)	0.2439

## Data Availability

All data are internal to our institution and not publicly available.
